# Face Management and Negative Strengthening: The Role of Power Relations, Social Distance, and Gender

**DOI:** 10.3389/fpsyg.2021.602977

**Published:** 2021-09-27

**Authors:** Nicole Gotzner, Diana Mazzarella

**Affiliations:** ^1^Cognitive Sciences, Department of Linguistics, University of Potsdam, Potsdam, Germany; ^2^Cognitive Science Centre, University of Neuchâtel, Neuchâtel, Switzerland

**Keywords:** conversational implicature, negation, politeness, social meaning, antonymy, adjectives

## Abstract

Negated gradable adjectives often convey an interpretation that is stronger than their literal meaning, which is referred to as ‘negative strengthening.’ For example, a sentence like ‘John is not kind’ may give rise to the inference that *John is rather mean*. Crucially, negation is more likely to be pragmatically strengthened in the case of positive adjectives (‘not kind’ to mean *rather mean*) than negative adjectives (‘not mean’ to mean *rather kind*). A classical explanation of this polarity asymmetry is based on politeness, specifically on the potential face threat of bare negative adjectives (Horn, 1989; Brown and Levinson, 1987). This paper presents the results of two experiments investigating the role of face management in negative strengthening. We show that negative strengthening of positive and negative adjectives interacts differently with the social variables of power, social distance, and gender.

## Introduction

The last few years have seen a growing interest in the experimental investigation of the role of social context in language comprehension. Social relations among interlocutors as well as social expectations in communication (e.g., politeness) have recently been experimentally manipulated to examine their effect on the interpretation of certain linguistic expressions, such as quantifiers and expressions of uncertainty (for reviews, see Holtgraves and Bonnefon, 2017; Holtgraves, 2019). The seminal paper of Bonnefon et al. (2009) opened up the question of the role of face management in the interpretation of utterances containing scalar terms like ‘some.’ Based on a series of experimental studies, Bonnefon and colleagues put forward the claim that the scalar term ‘some’ is less likely to be interpreted as conveying a pragmatically strengthened meaning (*some but not all*) when the utterance represents a threat to the positive social identity or ‘face’ of the addressee (‘Some people hated your poem’) than when it does not (‘Some people loved your poem’). These findings have been expanded - and debated - in subsequent studies that focused on further scalar expressions such as the connective *or* (Feeney and Bonnefon, 2012) and were investigated with distinct experimental techniques (i.e., reaction-times: Bonnefon et al., 2011; Mazzarella et al., 2018; and electrophysiology: Holtgraves and Kraus, 2018). As of today, though, this emerging experimental literature has not yet addressed the question of the role of face management in the interpretation of other linguistic expressions or constructions, beyond scalar and uncertainty expressions. This paper aims at filling this gap by looking at the interpretation of negated adjectives.

The interpretation of negated adjectives has long received the attention of philosophers, linguists and cognitive scientists due to its intuitive asymmetry (see e.g., Jespersen, 1917; Ducrot, 1973; Hoffmann, 1987; Horn, 1989; Colston, 1999; for more recent contributions see Giora et al., 2005; Krifka, 2007; Ruytenbeek et al., 2017; Gotzner et al., 2018). Consider a pair of antonymic adjectives like ‘kind’ and ‘mean.’ Crucially, the negation of the positive adjective (‘John is not kind’) is more likely to be interpreted as an affirmation of the antonym (*John is rather mean*) than the negation of the negative adjective (‘John is not mean’ interpreted as *John is rather kind*). This amounts to saying that positive adjectives are more likely to give rise to an inference called ‘negative strengthening’ compared to negative ones (Horn, 1989).

Interestingly for our purposes, it has been suggested that the use of negated positive adjectives (‘John is not kind’) to convey a negative interpretation (*John is rather mean*) can be seen as a politeness strategy by which the speaker may reduce the face threat toward the addressee carried by her speech act (Brown and Levinson, 1987; Horn, 1989; Levinson, 2000). ‘John is not kind’ is thus preferred to ‘John is mean’ as the former carries a reduced, less open, face threat (on the assumption that *kindness* is a desirable property). The addressee can unravel this reasoning and thus derive a strengthened interpretation of the negation. In the case of negated negative adjectives, or ‘double negatives,’ however, the affirmative statement often does not carry any potential face threat. For the running example, there is usually no reason relating to face management why speakers cannot directly say that ‘John is kind.’ Withholding this positive term may instead indicate that the situation does not quite match it (Levinson, 2000, p. 144). Thus, ‘John is not mean’ is interpreted as a middling term (e.g., *John is neither kind nor mean*) rather than licensing the inference to *John is kind*^1^.

The current work experimentally investigates the role of face management in the interpretation of positive and negative antonyms. It does so by looking into multiple sociological variables that calibrate the expected politeness level among interlocutors. In particular, we test the politeness explanation for the polarity asymmetry in two experiments by manipulating the social context in the following ways: (1) by inverting the power relation between the speaker and the hearer and (2) by manipulating their social distance. Based on the politeness explanation of negative strengthening, our main hypothesis is that these sociological variables interact with polarity in that they will mainly play a role in the interpretation of negated positive adjectives. Furthermore, we examine the role of participant gender and speaker gender in negative strengthening. As previous research has emphasized the relationship between face management and gendered communicative practices, we explore whether this relationship carries over to the pragmatic interpretation of negated adjectives.

Our paper is organized as follows. We first describe previous research on negative strengthening and introduce the framework of Politeness Theory by Brown and Levinson (1987). Second, we review key findings in the literature on language and gender and discuss their relevance for the present study. Third, we present our two experiments manipulating adjectival polarity and the sociological variables of power and distance. Experiment 1 focuses on power relations and Experiment 2 on the social distance between the speaker and the hearer. Finally, we discuss the results of our experiments in light of broader face management considerations based on both the speaker’s face and the hearer’s face and identify open questions for future research.

## Negated Adjectives and Social Context

### The Polarity Asymmetry of Negative Strengthening

The phenomenon of negative strengthening concerns the interpretation of negated antonymic adjectives. According to Horn (1989), negative strengthening arises when “under the right conditions, a formally contradictory negation not-F will convey a contrary assertion G” (Horn, 1989, p. 273). That is, under the right conditions, an utterance of ‘John is not kind’ (‘not-F’), which semantically encodes a meaning spanning from the *zone of indifference* between ‘kind’ and ‘mean’ to the contrary ‘mean,’ can be used to implicate that *John is rather mean* (‘G’) (for an alternative view, which models the gap between the extension of positive and negative antonyms as a pragmatic effect, see Krifka, 2007). When this is the case, the interpretation of the negated antonym (‘not kind’) is strengthened to convey *rather mean*. Crucially, according to this view, negative strengthening is an implicature, and, as such, it is a defeasible content. The utterance ‘John is not kind’ may lead the hearer to derive the implicature that *John is rather mean*, but this implicature can be defeated by a continuation like ‘But he is not mean either. Simply, don’t expect much support from him.’ Furthermore, the defeasibility of the strengthened meaning gives the speaker the possibility, if openly challenged, to retract it and to deny to have had the intention to convey such a meaning (see e.g., Lee and Pinker, 2010 on the deniability of indirect speech acts).

Research on negative strengthening has highlighted the following observation, which we refer to as the *polarity asymmetry of negative strengthening* (Bolinger, 1972; Ducrot, 1973; Brown and Levinson, 1987; Horn, 1989; Blutner and Solstad, 2000; Levinson, 2000): the negation of a positive polarity antonym (‘not kind’) is more likely to be strengthened than the negation of a negative polarity antonym (‘not mean’)^2^.

This generalization appeals to a notion of *polarity*, which allows us to distinguish between positive and negative antonyms. Polarity is traditionally defined based on the following three criteria (see Cruse, 1986). First, subjective judgments of desirability and undesirability (the so-called ‘evaluative polarity’): desirability maps onto positive polarity and undesirability maps onto negative polarity (Boucher and Osgood, 1969; Horn, 1989). For instance, ‘kind’ is desirable thus positive, ‘mean’ is undesirable thus negative. Second, the relevance of a certain dimension on the associated scale (the so-called ‘dimensional polarity’): the relevant dimension maps onto positive polarity. For instance, ‘tall’ and ‘short’ are associated with a scale of height, so ‘tall’ is positive and ‘short’ is negative (since the positive term is associated with a higher measurement value). Third, markedness based on morphological negation: markedness maps onto negative polarity. ‘Unhappy’ is morphologically marked by the negative affix un-, thus it is negative, ‘happy’ is unmarked, thus positive. While these three criteria often converge, there are also possible mismatches (see Cruse, 1986; Ruytenbeek et al., 2017 for a discussion).

The polarity asymmetry of negative strengthening has recently been confirmed by a rigorous experimental study. Ruytenbeek et al. (2017) employed both an acceptability judgment task and an inferential task to test participants’ interpretation of negated antonymic adjectives. The first task involved an indirect measure of negative strengthening based on acceptability judgments of sentences of the form ‘X is not P. Y is Q too,’ where P and Q represent an antonymic pair (e.g., in French *Paul n’est pas grand. Pierre aussi est petit*.). The second task allowed the collection of explicit inferential judgments by asking participants to judge the subject of the sentence on a continuous scale anchored at the antonyms P and Q (e.g., *Paul n’est pas grand* judged on a scale from *grand* to *petit*). Their results confirmed the expected polarity asymmetry: participants were more likely to strengthen the interpretation of negated positive antonyms than the interpretation of negated negative antonyms. They also showed that polarity interacts with morphological markedness, that is, the polarity asymmetry was greater for morphological pairs (containing negative morphemes such as ‘happy’ and ‘unhappy’) than for non-morphological pairs (involving lexical antonyms like ‘happy’ and ‘sad’). These results are in line with previous experiments by Colston (1999) and Fraenkel and Schul (2008). However, studies by Giora et al. (2005) and Paradis and Willners (2006) did not find an asymmetric pattern for the interpretation of positive and negative antonyms. Interestingly, these studies revealed that the interpretation of negated terms was dissimilar from their lexical antonyms for both positive and negative adjectives. For instance, the bare negative ‘sad’ was interpreted as conveying a lower degree of happiness than ‘not happy’ (as predicted by Krifka, 2007; see also Tessler and Franke, 2018; under review).

### Explaining the Polarity Asymmetry in Terms of Politeness

A traditional explanation of the polarity asymmetry of negative strengthening goes back to Horn (1989) and is framed in the context of Politeness theory (Brown and Levinson, 1987). According to Brown and Levinson, the interaction between speakers and hearers is typically regulated by face concerns, where face is defined as “the public self-image that every member [of a society] wants to claim for himself” (Brown and Levinson, 1987, p. 61, building on Goffman, 1967). Crucially, speakers might employ specific linguistic strategies - that Brown and Levinson call “politeness strategies” - to avoid or minimize a potential face loss. The speaker’s motivation to opt for a politeness strategy is a function of the level of face threat carried by their act (“weight of the face-threatening act”). Brown and Levinson (1987) identify three sociological variables influencing the calculation of the weight of a face-threatening act (Wx): power (P), distance (D), and ranking of imposition (R).


(1) Wx=P⁢(H,S)+D⁢(S,H)+Rx

P is the asymmetric social dimension of relative power that the hearer H has over the speaker S. The more powerful H is over S, the greater the weight of the face-threatening act. For instance, an utterance of ‘Your publication list is not rich’ would be more face-threatening when addressed to the Head of department by a student intern than the other way around. D is the symmetric social dimension of similarity/difference within which S and H stand for the purpose of the act x (typically based on the frequency of interaction and the exchange of social goods). The greater the distance between H and S, the greater the weight of the face-threatening act. Hence, ‘Your publication list is not rich’ is more face-threatening when addressed to a researcher you have just met at a conference than to your office mate. Finally, R is the ranking of imposition that the act x entails in a certain culture. The greater the imposition, the greater the weight of the face-threatening act. ‘Could you please proofread my grant proposal?’ is then more face-threatening than ‘Could you check my 250-word abstract?’. Overall, as the weight of the face-threatening act increases, speakers are more likely to adopt some politeness strategy. Importantly for our purposes, Brown and Levinson (1987) identify off-record strategies (including understatement) as politeness strategies that allow the speaker to avoid the responsibility of their communicative act by “leav[ing] it open to the addressee to decide how to interpret it” (1987, p. 211).

Drawing upon this framework, the negation of a positive antonym (‘not kind’) can be seen as a politeness strategy to mitigate the face threat carried by the alternative containing the other member of the antonymic pair (‘mean’). The threat might be a threat toward the face of the speaker, who wants to be perceived as benevolent and guarded (as in ‘John is not kind’), and/or toward the face of the addressee, who wants to be spared a direct criticism (as in ‘You are not kind’). As a result of politeness considerations, hearers may identify this strategy and consequently strengthen the interpretation of the negated positive antonym to convey its contrary. Crucially, though, the negation of a negative adjective (‘not mean’) does not make politeness a relevant consideration for the addressee. This is because the bare positive (‘kind’) would not elicit any potential face threat. It follows that politeness considerations facilitate negative strengthening in the case of negated positive adjective, but not in the case of negated negative adjective. Hence, the observed polarity asymmetry: “the relevant strengthening inference will tend to be favored in contexts [.] where there is some plausible reason to mask the speaker’s true opinion. These contexts characteristically involve [.] those gradable predications involving desirable properties, those whose denial would reflect undesirably on the subject, speaker, and or/addressee” (Horn, 1989, p. 334).

This traditional explanation of the polarity asymmetry calls for an experimental investigation to receive empirical support. A previous study from Gotzner et al. (2018) investigated the relationship between negative strengthening and scalar inferences^3^. In the context of this study, the authors collected participants’ ratings of the kindness/politeness of statements involving negated adjectives. This study did not find any evidence of a correlation between politeness ratings and degree of negative strengthening. However, as the authors acknowledge, “to discover effects of politeness, test sentences may have to be embedded within a rich conversational context in future studies and politeness may have to be manipulated directly in the experimental setup (Gotzner et al., 2018, p. 11). The current study takes up this challenge and experimentally investigates the role of face management in negative strengthening.

### Face Management and Gender

Before turning to our study, it is worth addressing the question of the relevance of gender to face management. Since the seminal work of Lakoff (1973), research on language and gender has focused on identifying specific gendered communicative practices as well as interpreting their significance in interaction. Early accounts argued for the existence of a relationship between linguistic features, such as hedges, tag questions, indirect requests and women’s subordinate social status in male-dominated environments. They thus identified power (or lack of) as the driving force of gendered communicative practices (see e.g., Lakoff, 1975). Later research, however, revealed the role of further dimensions, not reducible to status, in accounting for the observed language differences between women and men. For instance, Holmes’s (1984) and Cameron et al. (1988) empirical investigations challenged the idea that tag questions unequivocally express a lack of confidence or tentativeness and showed that their use served different functions: while men mainly used tag questions to express uncertainty, women tended to employ them as politeness devices to facilitate conversations or soften criticisms. Further examples of the prominence of solidarity-oriented behaviors in women language were found in the analysis of women’s gossip as well as women’s feedback to conversational partners (see e.g., Coates, 1988)^4^.

This view of women as supportive conversationalists is echoed by much research showing that, in many cultural and conversational contexts, women tend to be more polite than men (for an overview, see Chalupnik et al., 2017). For instance, Holtgraves and Yang (1992) experimentally demonstrate that women produce more polite requests than men (see also Baxter, 1984). Crucially, women are also *expected* to be more polite than men and are judged more severely than men when they fail to meet this expectation. In an experimental study focusing on alignment with the interlocutor’s opinion and compliance with their requests, Roberts and Norris (2016) found that a male delay was more tolerated than a female one: a female delay induced participant’s lower agreeableness ratings than an equivalent male delay.

These findings suggest that women and men exhibit (or are normatively expected to exhibit) differences in their face management. For the purpose of our study, it is thus relevant to investigate whether the pragmatic phenomenon of negative strengthening reveals any gender differences. For this reason, we included an exploratory analysis of the role of participant gender (Experiment 1 and Experiment 2) as well as of speaker gender (Experiment 2). Crucially, because most literature on gender and face-management focuses on gendered language production rather than comprehension, it is an open question whether similar patterns may emerge in pragmatic interpretation. If comprehension mirrored production, we would expect female participants to be more likely than male participants to interpret the use of a negated positive adjective as a politeness device and thus strengthen its negation. Concerning speaker gender, we would expect higher rates of negative strengthening when the utterance containing the negation of a positive adjective is attributed to female speakers. None of the previous studies on negative strengthening has looked into gender effects. Therefore, it is possible that some discrepancies across studies were caused by gender differences (e.g., the absence of a polarity asymmetry in the studies by Giora et al., 2005; Paradis and Willners, 2006).

## The Current Study

### Overview of Experiments

The aim of our experiments was two-fold. On the one hand, we aimed to assess the robustness of the polarity asymmetry and replicate the results obtained by Ruytenbeek et al. (2017). On the other hand, we aimed to investigate the role of face management with respect to this asymmetry. In two experiments, we tested the hypothesis that face management considerations affect the interpretation of negated positive adjectives in the following way: the greater the weight of the face-threatening act, the more likely the negation of the adjective will be pragmatically strengthened to convey its negative antonym. According to Brown and Levinson (1987), the weight of the face-threat depends, among other factors, on the power relation between speaker and hearer (P) and their social distance (D). For instance, if the speaker is in a less powerful position than the hearer, the speaker will be more likely to employ a politeness strategy to reduce the face threat carried by the speech act to be performed in a given context. In Experiment 1, we manipulated the power relation between speaker and hearer and in Experiment 2 the social distance between them. In addition, we analyzed participant gender as an exploratory analysis in Experiments 1 and 2, and we further manipulated the gender of the speaker in Experiment 2.

In each experiment, we embedded 20 negated antonym pairs (e.g., ‘not kind’ and ‘not mean’) in a context involving two dialog partners. Participants were asked to judge the speaker’s intended meaning on a 1-7 point Likert scale with 1 representing the adjective used in the original (negated) statement (e.g., ‘kind’) and 7 representing its antonym (e.g., ‘mean’). We pre-registered the experiments on OSF with the main prediction of an interaction between polarity and our sociological variables (power/distance) (Experiment 1^5^ : Experiment 2^6^).

### Experiment 1: Power Relations

#### Goals and Predictions

The first experiment investigates the role of power in negative strengthening for positive and negative adjectives by inverting the power relation between the speaker and the hearer (e.g., the professor talking to a student and vice versa). We use a 2 × 2 within-subject Latin Square design with polarity (positive, negative) and power (high power speaker, low power speaker) as factors. Our key dependent variable is the degree of negative strengthening. We measure the degree of negative strengthening by using a 7-point Likert scale anchored at the negated adjective (1) and its antonym (7).

Based on Brown and Levinson’s (1987) and Horn’s (1989) account of negative strengthening, we predict that the negation of a positive adjective is more likely to be pragmatically strengthened in the low power speaker condition than in the high power speaker condition. On the contrary, we expect no effect of power with respect to the pragmatic strengthening of negated negative adjectives. With regard to the comparison between positive and negative antonyms, we predict an interaction between polarity and power. Ruytenbeek et al. (2017) show a strength asymmetry in the negative strengthening of positive versus negative adjectives and attribute it to polarity. We predict that the asymmetry across positive and negative adjectives will be significantly stronger when the context makes politeness consideration relevant (low power speaker) than when it does not (high power speaker).

#### Methods

##### Participants

We recruited 60 participants with US IP addresses on Mechanical Turk (30 participants across two experimental lists). Participants were screened for native language and only included in the analysis if their self-reported native language was English. 34 men and 25 women participated in the study (one participant did not provide a response to the gender question). Their mean age was 37.15, with a standard deviation of 12.1 (age range 21 to 72). The experiment was conducted in accordance with the ethics policy of the Deutsche Forschungsgemeinschaft (DFG) under grant Nos. BE 4348/4-1 and GO 3378/1-1. Formal approval from an Ethics Committee is not required for adult studies according to national regulations. Participant’s consent was obtained at the start of the survey and their data were fully anonymized. The experiment lasted about 15 min and participants were paid 1 US Dollar in compensation.

##### Materials

We used the adjectives from Ruytenbeek et al. (2017) that had consistent polarity across different measures (markedness, evaluativity, and dimensionality). The items in the latter study were in French and we verified that English translation equivalent had the same polarity. In total, 20 adjectives pairs were used with their positive and negative antonyms occurring in a negated statement, thus totaling 40 critical items. The statements were embedded in a dialog between a speaker and hearer and preceded by a context sentence. Table 1 shows an example stimulus. The complete list of stimuli is available in Supplementary Appendix A.

**TABLE 1:** Example item for the adjective *fair* in Experiment 1 (positive polarity, low power speaker).

Context: At a staff gathering in the factory meeting room, the boss has presented the work-schedule he prepared for that day.
The boss asks an employee: “How do you find the schedule?”
The employee replies: “Your schedule is not fair”
According to the employee, the schedule is:
fair 1 2 3 4 5 6 7 unfair

The speaker who uttered the critical statement was either in high power position and the hearer in low power position (e.g., the boss responding to an employee’s question) or vice versa. We relied on the following three power relations: boss-employee, professor-student, editor-intern. The task of the participants was to indicate what the speaker wanted to communicate^7^. For example, in the sample stimulus, participants judged the extent to which - according to the speaker - the schedule is fair/unfair. Judgments were given on a 7-point Likert scale anchored at the negated adjective (1) and its antonym (7). Hence, we measure the degree of negative strengthening as a function of the likelihood with which the antonym of a pair is taken to be conveyed by the speaker’s utterance.

Our two factors, polarity and power, were all within-subject but spread across two different item lists in a Latin square design. Each participant saw 20 statements with positive and 20 statements with negative adjectives, rotated over power conditions. Hence, each participant completed 40 critical trials. The resulting overall number of critical observations was 2400. In addition to the critical items, participants were presented with 8 filler statements not involving negation, for example statements like ‘John is gorgeous’ (where the response scale was anchored the adjectives ‘gorgeous’ and ‘ugly’). The filler sentences also served as attention checks.

The experiment was programmed in HTML and run via Mturk’s in-built environment. The pre-registration form of the first experiment is available at the following link: (see text footnote 5)^8^.

##### Procedure

Participants read an instruction explaining the task with an example. They were told to judge what the individuals wanted to communicate. The running example was an adjective not used in the stimulus set (John asks Mary: How do I look? and Mary responds: You are not gorgeous). For each stimulus, the 1-7 point scale was anchored to the adjective used in the speaker’s statement (1) and its antonym (7). The instructions told participants to judge what the speaker wanted to convey in each dialog. Experimental trials and filler trials were randomized for each participant using an in-built randomization function.

#### Results

**FIGURE 1:**
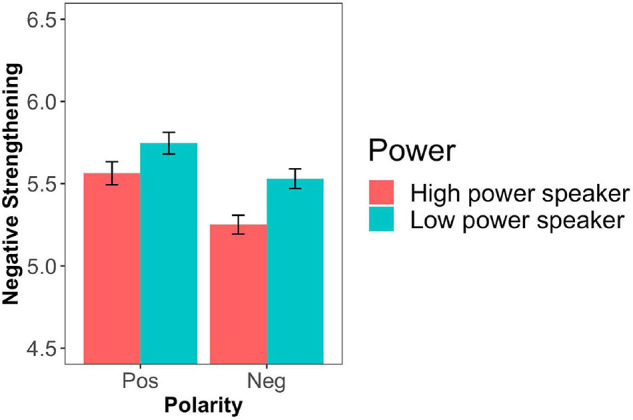
Mean degree of negative strengthening by adjective polarity and power condition (Experiment 1). Error bars represent ± 1 SEM.

The data were analyzed using R (version 3.6). We excluded four participants based on inconsistent responses in the filler trials (more than 50% responses not in line with the bare adjective used in the filler statements, i.e., a response of 5, 6, or 7). Figure 1 shows the mean responses by adjective polarity and power condition.

All results were analyzed with cumulative link mixed effects models using the function clmm() in the ordinal package (Christensen, 2018), which are more appropriate for Likert scales than linear mixed models^9^. We included the fixed factors power, polarity, their interactions as well as random intercepts for items and participants. All fixed factors were sum coded. The results of the model showed a main effect of polarity with positive adjectives involving a higher degree of negative strengthening than negative ones (*B* = −0.35, *SE* = 0.04, *z* = −8.27, *p* < 0.0001). This finding replicates the polarity asymmetry discussed in previous work (e.g., Ruytenbeek et al., 2017). In addition, there was a main effect of power with a higher degree of negative strengthening for speakers in a low power position than in a high power position (*B* = −0.2, *SE* = 0.04, *z* = −4.76, *p* < 0.0001). The interaction between polarity and power was not significant (*p* = 0.5). A summary of the model is presented in Table 2.

**TABLE 2:** Summary of cumulative link mixed effects model including the sum-coded fixed effects power and polarity (Experiment 1).

	**Estimate**	***SE***	***z*-value**	***p*-value**
Polarity	−0.35461	0.04287	−8.272	0.0001
Power	−0.20108	0.04228	−4.756	0.0001
Polarity: power	−0.17623	0.27286	−0.646	0.518

**FIGURE 2:**
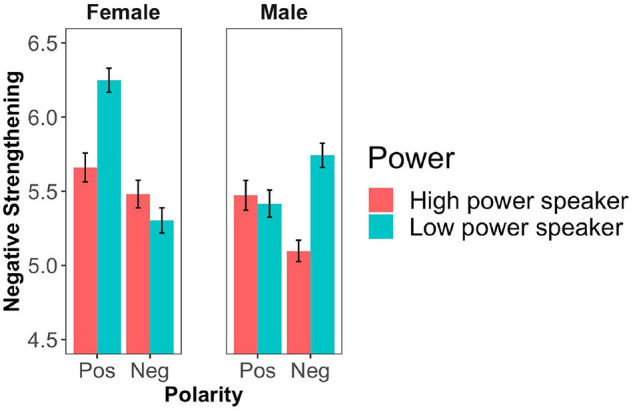
Mean degree of negative strengthening by adjective polarity, power and participant gender (Experiment 1). Error bars represent ± 1 SEM.

As an exploratory analysis, we computed a model with participant gender as an additional treatment-coded variable. Female participants were chosen as the reference level based on the previous literature suggesting that women tend to be more polite than men (for an overview, see Chalupnik et al., 2017). The model again revealed main effects for polarity and power. Further, there was an interaction between participant gender and polarity (*B* = 0.59, *SE* = 0.087, *z* = 6.81, *p* < 0.0001) as well as a tendency toward a three-way interaction of polarity, power and participant gender (*B* = −0.098, *SE* = 0.54, *z* = −1.79, *p* = 0.07). The interaction between participant gender and polarity reveals a larger polarity asymmetry for female participants than male participants. Furthermore, the tendency toward a three-way interaction with power indicates that female participants displayed an effect of power for positive adjectives but not negative adjectives while male participants displayed the opposite pattern. The means across conditions and participant gender are displayed in Figure 2 and the results of the cumulative link mixed effects model are presented in Table 3.

**TABLE 3:** Summary of cumulative link mixed effects model including the sum-coded fixed effects power, polarity and treatment-coded fixed effect participant gender with females as the reference level (Experiment 1).

	**Estimate**	***SE***	***z*-value**	***p*-value**
Polarity	−0.69527	0.06782	−10.252	0.0001
Power	−0.18941	0.06654	−2.847	0.00442
Participant gender male	−0.50794	0.54546	−0.931	0.35175
Polarity: power	0.39707	0.40973	0.969	0.33249
Polarity: participant gender male	0.59673	0.08761	6.812	0.0001
Power: participant gender male	−0.10074	0.08714	−1.156	0.24767
Polarity: power: participant gender male	−0.97942	0.54605	−1.794	0.07287

#### Discussion

Experiment 1 showed an asymmetry of negative strengthening for positive and negative adjectives. That is, negated positive terms were more likely to be strengthened than negated negative terms, replicating the findings of Ruytenbeek et al. (2017)^10^. In addition, we found a main effect of power: negative strengthening was more likely to occur when the speaker was in a low power position as opposed to a high power position. Contrary to the main prediction, we did not find an interaction between polarity and power. That is, independently of the polarity of the adjective, participants were more inclined to interpret low power speakers’ utterances as indirect affirmations of the contrary.

As previous research suggests the existence of gender differences in face-management, we conducted an exploratory analysis with gender as a binary factor. We found an interaction between polarity and participant gender with female participants displaying a greater degree of negative strengthening for positive compared to negative adjectives. Furthermore, there was marginal three-way interaction indicating that female participants showed a tendency for a stronger effect of the power manipulation for positive adjectives, which goes in the direction of the predicted interaction. Male participants, in turn, were mainly affected by the power manipulation for negative adjectives. That is, male participants were more likely to strengthen a negated negative statement when the speaker was in the low power position.

### Experiment 2: Social Distance

#### Goals and Predictions

The second experiment manipulated social distance with the speaker and the hearer being either close friends (low social distance) or having just met (high social distance). The professions used in Experiment 1 were replaced with common names. All dialogs were between same-gender names, with half of them including stereotypically female names and the other half stereotypically male names (speaker gender manipulation). Our main prediction, based on Brown and Levinson (1987) and Horn (1989), was again an interaction between polarity and distance. That is, participants should be more likely to strengthen the negation of positive adjectives when the addressee is socially distant than when the addressee is socially close. Since Experiment 1 showed a trend for the predicted interaction across polarity and power for female participants, we included participant and speaker gender in our analysis.

#### Methods

##### Participants

We recruited another set of 60 participants with US IP addresses on Mechanical Turk (30 participants across two experimental lists). Participants were screened for native language and only included in the analysis if their self-reported native language was English. One participant’s native language was Italian and the data were therefore excluded from further analyses. The remaining 59 participants were 30 men and 29 women with a mean age of 37.18 and a standard deviation of 11.5 (age range: 22 to 65). The experiment was conducted in accordance with the ethics policy of the Deutsche Forschungsgemeinschaft (DFG) under grant Nos. BE 4348/4-1 and GO 3378/1-1. Formal approval from an Ethics Committee is not required for adult studies according to national regulations. Participant’s consent was obtained at the start of the survey and their data were fully anonymized. The experiment lasted about 15 min and participants were paid 1 US Dollar in compensation.

##### Materials

The materials were the same as in Experiment 1 but we replaced noun phrases with common names (e.g., John and Paul). Social distance was manipulated by describing the two characters as either friends (low social distance) or having just met (high social distance). Dialogs were always between speakers of the same gender and we created items in which either two men or two women interacted (based on stereotypical names). We used the most common American English names for men and women. Table 4 shows an example stimulus. The complete list of stimuli is available in Supplementary Appendix B. The pre-registration form of the second experiment is available at the following link: (see text footnote 6)^11^.

**TABLE 4:** Example item for the adjective *fair* in Experiment 2 (negative polarity, high social distance, female speakers).

Context: Sue and Mary just started working in the same company. At a staff gathering in the factory meeting room, Mary has presented the work-schedule she prepared for that day.
Mary asks Sue: “How do you find the schedule?”
Sue responds: “Your schedule is not unfair”
According to Sue, the schedule is:
unfair 1 2 3 4 5 6 7 fair

##### Procedure

The procedure was the same as that of Experiment 1.

#### Results

**FIGURE 3:**
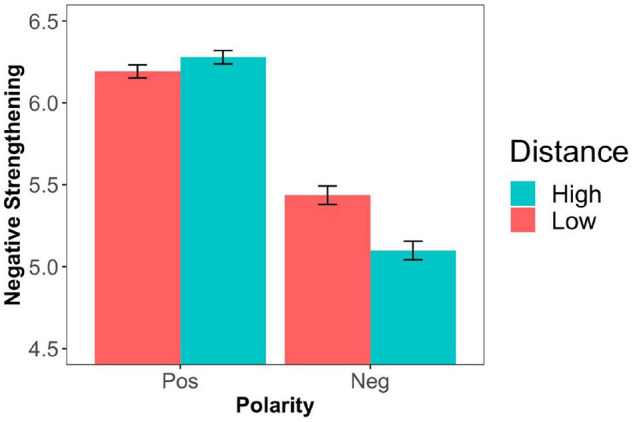
Mean degree of negative strengthening by adjective polarity and social distance (Experiment 2). Error bars represent ± 1 SEM.

**FIGURE 4:**
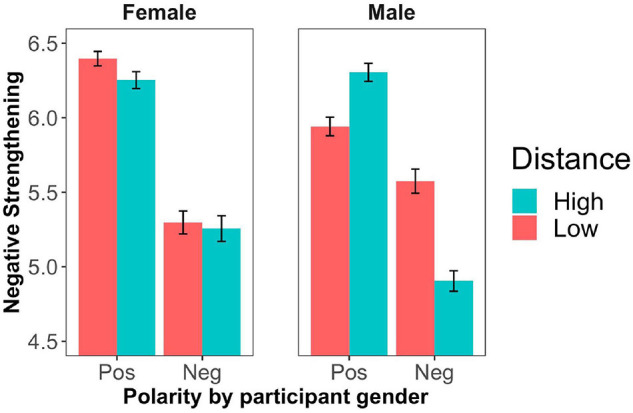
Mean degree of negative strengthening by adjective polarity, social distance and participant gender (Experiment 2). Error bars represent ± 1 SEM.

**FIGURE 5:**
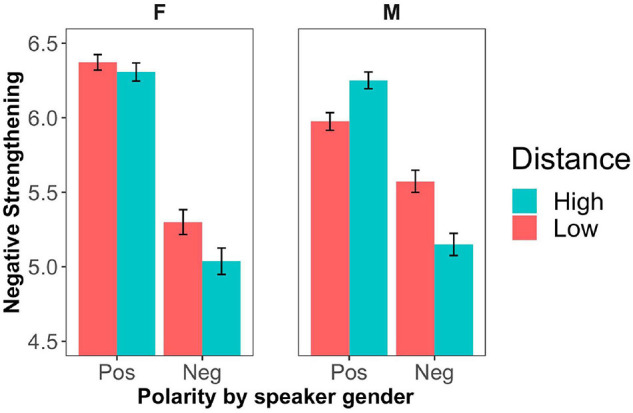
Mean degree of negative strengthening by adjective polarity, social distance and speaker gender as manipulated in Experiment 2 (labeled with ‘F’ for female and ‘M’ for male). Error bars represent ± 1 SEM.

Four participants were excluded from further analyses for giving inconsistent responses in filler trials (more than 50% responses not in line with the bare adjective used in the filler statements, i.e., a response of 5, 6, or 7). Figure 3 shows the mean ratings across polarity and social distance conditions. In Figure 4, we present the results across participant gender and in Figure 5 across speaker gender.

We first ran a cumulative link model involving the factors social distance, polarity, and their interaction as well as a random intercept for participants and items. Again, we replicated the polarity effect (*B* = −1.05, *SE* = 0.048, *z* = −21.94, *p* < 0.0001). The main effect of social distance was not significant and neither was the interaction between polarity and distance, as presented in Table 5^12^.

**TABLE 5:** Summary of cumulative link mixed effects model including the sum-coded fixed effects polarity and social distance (Experiment 2).

	**Estimate**	***SE***	***z*-value**	***p*-value**
Polarity	−1.04967	0.04784	−21.94	0.0001
Distance	−0.05998	0.0427	−1.405	0.16
Polarity: distance	−0.29434	0.22804	−1.291	0.197

Further, we ran a second model including the additional treatment-coded variable participant gender (female as reference level). In addition to the main of effect of polarity (*B* = −1.15, *SE* = 0.06, *z* = −17,7, *p* < 0.0001), this model revealed an interaction between polarity and participant gender (*B* = 0.20, *SE* = 0.09, *z* = 2.39, *p* < 0.05), showing again that female participants displayed a greater polarity asymmetry compared to male participants. Further, there was three-way interaction between polarity, distance and participant gender (*B* = −0.09, *SE* = 0.04, *z* = −2.01, *p* < 0.05). This interaction indicated that female participants were less affected by the social distance manipulation and that male participants differentially strengthened positive and negative terms depending on social distance. The detailed results are presented in Table 6.

**TABLE 6:** Summary of cumulative link mixed effects model including the sum-coded fixed effects polarity, social distance, and treatment-coded fixed effect participant gender with females as the reference level (Experiment 2).

	**Estimate**	***SE***	***z*-value**	***p*-value**
Polarity	−1.15166	0.06447	−17.865	0.0001
Distance	−0.05732	0.06017	−0.953	0.3407
Participant gender male	−0.38879	0.43874	−0.886	0.3755
Polarity: distance	0.12415	0.30197	0.411	0.681
Polarity: participant gender male	0.20435	0.08546	2.391	0.0168
Distance: participant gender male	−0.01142	0.08559	−0.133	0.8939
Polarity: distance: participant gender male	−0.88616	0.43893	−2.019	0.0435

Finally, we ran a model with polarity, social distance, participant gender and speaker gender (i.e., our manipulated variable of the dialog partner’s stereotypical names) as factors. This model showed main effects of polarity (*B* = −1.43, *SE* = 0.09, *z* = −15.7, *p* < 0.0001), social distance (*B* = 0.24, *SE* = 0.09, *z* = 2.79, *p* < 0.01), an interaction between polarity and speaker gender (*B* = 0.52, *SE* = 0.12, *z* = 4.3, *p* < 0.0001), an interaction between social distance and speaker gender (*B* = −0.36, *SE* = 0.12, *z* = −2.96, *p* < 0.01), an interaction between polarity and participant gender (*B* = 0.24, *SE* = 0.12, *z* = 1.98, *p* < 0.05) as well as three-way interactions between polarity, distance and speaker gender (*B* = 0.28, *SE* = 0.13, *z* = 2.2, *p* < 0.05) and polarity, distance and participant gender (*B* = 0.96, *SE* = 0.45, *z* = 2.13, *p* < 0.05) (see Table 7). For male names, negative strengthening was more likely for socially close dialog partners when the adjective was negative, but for socially distant dialog partners when the adjective was positive. Dialogs involving female names also showed a greater polarity than those with male names, as evident in the interaction between polarity and speaker gender. Speaker gender and participant gender did not show any interactions.

**TABLE 7:** Summary of cumulative link mixed effects model including the sum-coded fixed effects polarity, social distance, participant gender, and speaker gender with female participants and female names as the reference level (Experiment 2).

	**Estimate**	***SE***	***z*-value**	***p*-value**
Polarity	−1.42778	0.0909	−15.707	0.0001
Distance	0.24481	0.08782	2.787	0.00531
Speaker gender male	−0.1274	0.14441	−0.882	0.37768
Participant gender male	−0.42005	0.454	−0.925	0.35485
Polarity: distance	−0.2582	0.31353	−0.824	0.4102
Polarity: speaker gender male	0.52103	0.12063	4.319	0.0001
Distance: speaker gender male	−0.36401	0.12316	−2.955	0.00312
Polarity: participant gender male	0.24358	0.12295	1.981	0.04757
Distance: speaker gender male	−0.12515	0.12306	−1.017	0.30919
Speaker gender male: participant gender male	0.05489	0.17031	0.322	0.74723
Polarity: Distance: speaker gender male	0.28304	0.12872	2.199	0.02788
Polarity: distance: participant gender male	0.9655	0.45425	2.125	0.03355
Polarity: speaker gender male: participant gender male	−0.07521	0.17027	−0.442	0.65873
Distance: speaker gender male: participant gender male	0.2608	0.17038	1.531	0.12585
Polarity: distance: speaker gender male: participant gender male	−0.14146	0.17036	−0.83	0.40634

#### Discussion

In our second experiment, we replicated the polarity asymmetry of negative strengthening as well as the interaction between polarity and participant gender. As in Experiment 1, negated positive adjectives were more likely to be strengthened than negated negative adjectives and this asymmetry was stronger for female participants than for male participants. Furthermore, we found that social distance had distinct effects for positive and negative adjectives, depending on participant/speaker gender. For male participants/speakers, negative strengthening was more likely to occur when dialog partners were socially close if the adjective was negative (‘not mean’ to mean *rather kind*). However, when the adjective was positive, in line with our prediction, negative strengthening was more likely to occur (‘not kind’ to mean *rather mean*) when dialog partners were socially distant. In contrast with this, for female speakers/participants, there was no effect of the distance manipulation for positive adjectives. When the adjective was negative, female speakers (but not female participants) also gave rise to more negative strengthening for socially close dialog partners.

## General Discussion

Across our two experiments, we examined the role of two of the sociological variables identified by Brown and Levinson (1987), that is, power (P) and social distance (D), on the interpretation of negated antonyms. Furthermore, we investigated the existence of possible gender effects by looking both at participant gender and speaker gender.

Across our experiments we found two clear and consistent patterns: (i) the existence of a polarity asymmetry in the interpretation of negated adjectives; (ii) an interaction between adjectival polarity and participant gender. The first finding represents an important replication of Ruytenbeek et al.’s (2017) results by using contextually richer scenarios (as opposed to decontextualized sentences) and confirms the reliability of our adapted paradigm. The second finding reveals that the polarity asymmetry of negative strengthening is modulated by participant gender: female participants display a stronger polarity asymmetry than male participants, as evident in consistent interactions between polarity and participant gender across our two experiments.

Furthermore, we showed that power and social distance both had an effect on the interpretation of negated antonyms. However, in contrast with our main prediction, their effect differed in the following way. With respect to power, Experiment 1 showed that the greater the power of the hearer over the speaker, the stronger was the degree of negative strengthening (with an interesting tendency for an interaction between polarity and participant gender, as revealed by our exploratory analysis). With respect to social distance, Experiment 2 revealed the following interaction with polarity and participant gender. For male participants, the greater the social distance between the speaker and the hearer, the stronger was the degree of negative strengthening for positive adjectives. Furthermore, the smaller the social distance between the speaker and the hearer, the stronger was the degree of negative strengthening for negative adjectives.

Overall, the results do not support a straightforward explanation of the polarity asymmetry of negative strengthening based on politeness considerations, as the one suggested by Brown and Levinson (1987) and Horn (1989). This traditional explanation would have predicted an interaction between polarity and the social variables of power and distance, such that greater negative strengthening for positive adjectives should have occurred when the speaker was in a low power position compared to the addressee (Experiment 1) or socially distant to the addressee (Experiment 2). Crucially, our results reveal a more complicated picture, one in which the social variable of gender plays an important role. Indeed, the expected effects of power and distance on negative strengthening for positive adjectives selectively appeared only when gender was factored in.

In Experiment 1, female participants – but not male participants – tended to be more likely to strengthen the negation of a positive adjective when the speaker was in a low power position compared to a high power position. That is, when confronted with an utterance like ‘Your schedule is not fair,’ which represents a potentially face-threatening act, female participants tended to interpret it as a function of the relative power of the hearer over the speaker. They were more likely to strengthen their interpretation toward *Your schedule is unfair* when the speaker was an employee and the addressee the boss, than the other way around.

In Experiment 2, male participants – but not female participants – were more likely to strengthen the negation of a positive adjective when the speaker was in a high distance relationship with the hearer than when they were socially close. That is, when confronted with an utterance like ‘Your schedule is not fair,’ male participants were more likely to attribute the intention to communicate a strengthened interpretation (*Your schedule is rather unfair*) to socially distant speakers than to socially close ones.

Interestingly, our results suggest that female and male individuals might differ in their attribution of the intention to minimize a face-threat when the interaction involves the expression of evaluations via negated adjectives (‘Your schedule is not fair’). First of all, the results indicated that female participants are more likely than male participants to strengthen the negation of a positive adjective (‘Your schedule is not fair’ to mean *Your schedule is rather unfair*). This suggests that female participants are more prone than male participants to take the negation of a positive adjective as an indirect negative evaluation. Such a gendered interpretative behavior is in line with previous literature suggesting that women are more likely than men to appeal to standard politeness strategies such as indirectness (Holtgraves and Yang, 1992). As suggested by Brown (1980), women are “more sensitive from moment to moment to the face-threatening potential of what they are saying and modify their speech accordingly” (Brown, 1980, p. 131). This parallelism indicates that not only do women rely on polite indirectness more often than men, but they are also more likely to attribute this strategy to speakers in potentially face-threatening situations. Interestingly, data from Experiment 2 highlight that the strength of the polarity asymmetry of negative strengthening also varies as a function of speaker gender: the negation of positive adjectives is more likely to be strengthened when the utterance is attributed to a female speaker. This may indicate that, consistently with findings from Roberts and Norris (2016), participants expected female speakers to be more polite than male speakers, and thus interpreted the use of negation in utterances like ‘Your schedule is not fair’ as polite indirectness to communicate that *Your schedule is rather unfair*.

Furthermore, our data reveal gendered interpretations of negated antonyms as a function of both power and social distance. For instance, when looking at the negative strengthening of negated positive adjectives, we found that that female participants were more likely to attribute face-saving intentions as a function of power, while male participants as a function of social distance. It is worth noticing that these results suggest different sensitivities to face-threat across genders in relation to the interpersonal nature of the context (for a similar conclusion, see Holtgraves and Yang, 1992). As suggested by Holtgraves and Yang (1992), this may be the result of differences in the *perceptions* of the situation on the power and social distance dimensions, and/or differences in the *weighting* of power and social distance. This suggests that a full appreciation of the formula provided by Brown and Levinson to describe the way in which power and social distance influence the weight of a face-threatening act, Wx = P (H,S) + D (S,H) + Rx, cannot overlook some important dimensions of variation, such as cultural patterns that hold for specific groups or social categories. Among these, the gender of an individual appears to be linked to normatively stabilized expectations about the way in which they will perceive or weigh a face-threatening act, thus giving rise to regularities in interactional strategies.

Finally, there is one interesting finding that deserves further attention. Across both experiments, male participants showed greater variability in their interpretation of negated negative adjectives, or double negatives, as a function of the interpersonal context. When confronted with an utterance like “Your schedule is not unfair,” male participants derive a strengthened interpretation (*Your schedule is rather fair*) in the following two circumstances: when the speaker is in a relatively low power position and when the speaker is socially close to the hearer. This unexpected result opens up the question of the role of double negatives in interactions and their gendered interpretation. While this is ultimately an empirical question, we suggest that future research might benefit from focusing on considerations about the speaker’s face. As Brown and Levinson have argued at length, face-management ordinarily involves considerations about both speaker and hearer face. In his discussion of negative strengthening, Horn mentions that in Western cultures there is sometimes a taboo to state positive emotions directly and to show excessive enthusiasm (Horn, 1989, p. 359). Because of this, speakers may use a weak statement (‘Your schedule is not unfair’) as a “studied modesty of expression” (Stoffel, 1901, p. 126) in order to safeguard their face, e.g., in order not to appear as overly positive. Indeed, there is some independent evidence that men are expected to temper their positivity to preserve their perceived power (see Sattel, 1983). This kind of face-management concern might have played a role in the pattern of interpretation observed for male participants. Male participants might assume that the speaker will not want to appear overly positive when complimenting a more powerful addressee (hence avoiding being perceived as motivated by the opportunistic desire of pleasing the addressee) or a friend (hence avoiding showing overt admiration). This suggestion fits well with established gender differences in paying compliments (see e.g., Holmes, 1988; Herbert, 1998). Furthermore, it has been noted that certain uses of double negatives convey an interpretation that is stronger than the bare positive as a form of polite understatement (Lyons, 1977; Horn, 1991; Levinson, 2000; Krifka, 2007). An example of this is the use of ‘not bad’ to mean *very good* (for the role of prosody in eliciting this interpretation see Bolinger, 1972, p. 116). Future work is needed to distinguish the role of the speaker’s face and the hearer’s face in negative strengthening, as well as their interaction with gender. Overall, our findings fit well with the theoretical assumptions of recent modeling of polite speech (see e.g., Yoon et al., 2017, to appear), which suggest that politeness emerges from competing social goals. By applying a model comparison approach, these studies show that, over and beyond the informative utility of the communicative act, speakers rely on considerations of pro-social as well as self-presentational utilities when designing their utterances in potentially face-threatening contexts. The role of self-presentational considerations in the adoption of face-management strategies targeting the speaker’s face represents a topical issue for future experimental research. In future work, we will extend the current manipulations to the area of language production.

Another productive line of future research concerns the investigation of different notions of polarity. An experiment by Mazzarella and Gotzner (2021) revealed that the standard polarity asymmetry in negative strengthening holds even in contexts in which the face-threatening potential of positive and negative utterances is reversed. These findings indicate a role of adjective polarity that is somewhat independent of face management considerations. However, all previous studies in this area have tested adjective pairs that are consistently positive or negative in terms of evaluative, dimensional polarity and markedness. We propose that investigating polarity mismatches (e.g., ‘dirty,’ which is evaluatively negative but dimensionally positive) will provide crucial insights into the mechanisms underlying negative strengthening and its polarity asymmetry.

In sum, the results of our study indicate that while face-management considerations have an impact on the interpretation of negated adjectives, but this impact is not limited to the interpretation of positive adjectives. Both positive and negative negated adjectives might undergo a process of negative strengthening as a function of the power relation and social distance among the interlocutors. This suggests that the interplay between face-management and negative strengthening is more complex than previously assumed and it opens up new lines for future research.

## Conclusion

The present study investigated the role of face management in negative strengthening by manipulating the social context in two different ways: via the manipulation of the power relation between the dialog partners (Experiment 1) and their social distance (Experiment 2). Furthermore, it investigated the presence of gender effects by manipulating the identity of the speaker and examining the interpretative behaviors of female and male participants. The study provided empirical support to the polarity asymmetry of negative strengthening, in line with results from Ruytenbeek et al. (2017). In both experiments, we observed a significant effect of polarity on the interpretation of negated adjectives: positive adjectives were more likely to be negatively strengthened than negative adjectives. Crucially, though, we found that the social context affected the degree of negative strengthening for both positive and negative adjectives. This is in contrast with the prediction based on Brown and Levinson (1987) and Horn (1989) that social context should affect the interpretation of positive adjectives only. While Horn (1989) does consider the role of a taboo to state positive emotions directly in certain cultures, the main explanation of the polarity asymmetry concerned the face-threatening potential of bare negatives. What is more, this standard explanation did not anticipate the complex interactions between different sociological variables. Overall, our results indicate that negative strengthening is the result of wider face management considerations, which might concern both the speaker’s intention to mitigate the threat toward the face of the addressee and the speaker’s intention to save their own face.

The present study also reveals the existence of gendered expectations about the use and meaning of linguistically conveyed evaluations via negated adjectives. As gender represents an important attribute of an individual’s identity, these results confirm the interconnection between face-management and identity, whose importance has been foregrounded by more recent approaches in politeness research (see e.g., Spencer-Oatey, 2009). Furthermore, research on the relationship between language and gender has primarily focused on language production. Our results thus contribute to extend this investigation to the domain of pragmatic interpretation.

Finally, our study broadens the array of pragmatic phenomena that have been investigated with the aim of addressing the question of the interface between politeness and pragmatic inference (see e.g., Bonnefon et al., 2009; Feeney and Bonnefon, 2012; Mazzarella et al., 2018). By focusing on negative strengthening, it enriches our understanding of the way in which language interpretation depends on social context.

## Data Availability Statement

The data of Experiment 1 are available at https://osf.io/67he9/ and those of Experiment at https://osf.io/3kqfv/.

## Ethics Statement

The experiment was conducted in accordance with the ethics policy of the Deutsche Forschungsgemeinschaft (DFG) under grant Nos. BE 4348/4-1 and GO 3378/1-1. Formal approval from an Ethics Committee is not required for adult studies according to national regulations. Participant’s consent was obtained at the start of the survey and their data were fully anonymized.

## Author Contributions

NG implemented the experiments, performed the statistical analyses, and wrote the first draft of Section “The Current Study” and parts of Section “Introduction.” DM wrote the first draft of Sections “Introduction, Negated Adjectives and Social Context, General Discussion, and Conclusion”. Both authors contributed to the conception and design of the study and edited the manuscript, and read and approved the final submitted version.

## Conflict of Interest

The authors declare that the research was conducted in the absence of any commercial or financial relationships that could be construed as a potential conflict of interest.

## Publisher’s Note

All claims expressed in this article are solely those of the authors and do not necessarily represent those of their affiliated organizations, or those of the publisher, the editors and the reviewers. Any product that may be evaluated in this article, or claim that may be made by its manufacturer, is not guaranteed or endorsed by the publisher.

## Footnotes

1 But see Horn (1989) and Krifka (2007) who point out that certain uses of double negatives do imply the positive form. We return to this point in the General discussion.

2 The downtoning effect of negation, thanks to which double negatives appear to have a weakened attenuated sense, is known since Jespersen (1917).

3 See also Leffel et al. (2019) who show that relative adjectives like ‘tall’ but not minimum standard adjectives like ‘late’ are negatively strengthened (in the ‘not very’ construction, e.g., ‘John is not very tall’).

4 For an extensive overview of the interdisciplinary research on language and gender, see Eckert and McConnell-Ginet (2013).

5
https://osf.io/d5e6u

6
https://osf.io/knrdz

7 In contrast to Ruytenbeek et al.’s (2017) study, our test question involved the explicit attribution of the intended implication to the speaker.

8 We had originally planned to run linear models as the study by Ruytenbeek et al. (2017) did. Upon closer inspection, we noticed that, in this study, the response variable was re-coded into a binary variable. In the meantime, cumulative link mixed effects models have become the standard to analyze ordinal data. Since such models are more appropriate for ordinal data than linear models, we decided to analyse our data with the former kind.

9 The function clmm() is the more recent variant of clmm2(), allowing for the implementation of multiple random effects. However, at the time of running our experiments and analyzing the data, no random slopes were implemented for ordinal models (see Christensen, 2018).

10 Following Ruytenbeek et al. (2017), we also looked at the extent to which morphologically complex pairs (e.g., *happy-unhappy*) triggered a greater degree of negative strengthening compared to simple antonymic pairs (e.g., *happy-sad*). The additional model revealed an interaction between the factors polarity and morphological complexity in the predicted direction (*B* = −0.12, *SE* = 0.042, *z* = −2.81, *p* < 0.01), thus replicating the results of Ruytenbeek et al. (2017).

11 The pre-registration reports the manipulation of speaker gender. As the main hypotheses derived from the politeness explanation concerned the interaction of polarity and social distance, only these were considered in our pre-registered analysis plan.

12 Additionally, we computed a model to see whether the polarity asymmetry was greater for morphologically complex antonym pairs vs. simple ones and we again found a significant interaction (*B* = −0.36, *SE* = 0.043, *z* = −8.46, *p* < 0.0001).
