# Supplementary material for: Face Management and Negative Strengthening: The Role of Power Relations, Social Distance, and Gender
Source: Front Psychol. 2021 Sep 27;12:602977. doi: 10.3389/fpsyg.2021.602977 (PMC8502883; doi:10.3389/fpsyg.2021.602977)
Supplement: Supplementary file 1 [file Table_1.pdf]

APPENDIX A
List of stimuli for Experiment 1

Antonymic pair 1: CERTAIN/UNCERTAIN

Condition: Low power speaker
The company is negotiating a deal with an important customer. The boss is in charge of getting the deal through. The boss asks an employee: “What do you think of the deal?”
The employee responds: “Your deal is not certain”/ “Your deal is not uncertain”

Condition: High power speaker
The company is negotiating a deal with an important customer. An employee is in charge of getting the deal through. The employee asks the boss: “What do you think of the deal?”
The boss responds: “Your deal is not certain”/ “Your deal is not uncertain”

Antonymic pair 2: LUCKY/UNLUCKY

Condition: Low power speaker
Following renovations, the boss has been assigned a new office room. The boss asks an employee: “How do you find my office room?”
The employee responds: “You were not lucky”/ “You were not unlucky”

Condition: High power speaker
Following renovations, an employee has been assigned a new office room. The employee asks the boss: “How do you find my office room?”
The boss responds: “You were not lucky”/ “You were not unlucky”

Antonymic pair 3: ACCURATE/INACCURATE

Condition: Low power speaker
The newspaper prepares a special issue on the upcoming election. The editor has written a feature on the leading candidate. The editor asks the intern: “What do you think of the feature?”
The intern responds: “Your feature is not accurate”/ “You feature is not inaccurate”

Condition: High power speaker
The newspaper prepares a special issue on the upcoming election. An intern has written a feature on the leading candidate. The intern asks the editor: “What do you think of the feature?”
The editor responds: “Your feature is not accurate”/ “You feature is not inaccurate”

Antonymic pair 4: HAPPY/UNHAPPY

Condition: Low power speaker

At a meeting with an important client, the boss has presented his new marketing strategy. The boss asks an employee: “How did you find the client's reaction?”
The employee responds: “The client was not happy”/ “The client was not unhappy”

<u>Condition: High power speaker</u>
At a meeting with an important client, an employee has presented his new marketing strategy. The employee asks the boss: “How did you find the client's reaction?”
The boss responds: “The client was not happy”/ “The client was not unhappy”

Antonymic pair 5: INTERESTING/UNINTERESTING

<u>Condition: Low power speaker</u>
In history class, the professor has shown a video about the French Revolution. The professor asks a student: “What do you think of the video?”
The student responds: “The video is not interesting”/ “The video is not uninteresting”.

<u>Condition: High power speaker</u>
In history class, a student has shown a video about the French Revolution. The student asks the teacher: “What do you think of the video?”
The teacher responds: “The video is not interesting”/ “The video is not uninteresting”.

Antonymic pair 6: FAIR/UNFAIR

<u>Condition: Low power speaker</u>
At a staff gathering in the factory meeting room, the boss has presented the work schedule he prepared for that day. The boss asks an employee: “How do you find the schedule?”
The employee responds: “Your schedule is not fair”/ “Your schedule is not unfair”

<u>Condition: High power speaker</u>
At a staff gathering in the factory meeting room, an employee has presented the work schedule he prepared for that day. The employee asks the boss: “How do you find the schedule?”
The boss responds: “Your schedule is not fair”/ “Your schedule is not unfair”

Antonymic pair 7: POLITE/IMPOLITE

<u>Condition: Low power speaker</u>
The newspaper would like to interview the leading candidate of the upcoming election. The editor writes an email to request a meeting. The editor asks an intern: “What do you think of my email?”
The intern responds: “Your email is not polite”/ “Your email is not impolite”

<u>Condition: High power speaker</u>

The newspaper would like to interview the leading candidate of the upcoming election. An intern writes an email to request a meeting. The intern asks the editor: “What do you think of my email?”
The editor responds: “Your email is not polite”/ “Your email is not impolite”

Antonymic pair 8: POSSIBLE/IMPOSSIBLE

Condition: Low power speaker
The boss is behind with the working schedule. The prototype needs to be delivered to the client by the end of the week. The boss asks an employee: “What do you think about extending the deadline?”
The employee responds: “An extension is not possible”/ “An extension is not impossible”

Condition: High power speaker
An employee is behind with the working schedule. The prototype needs to be delivered to the client by the end of the week. The employee asks the boss: “What do you think about extending the deadline?”
The boss responds: “An extension is not possible”/ “An extension is not impossible”

Antonymic pair 9: SATISFACTORY/UNSATISFACTORY

Condition: Low power speaker
At the yearly meeting of the press association, the editor is giving a talk. The editor asks an intern: “What do you think of my performance?”
The intern responds: “Your performance was not satisfactory”/ “Your performance was not unsatisfactory”

Condition: High power speaker
At the yearly meeting of the press association, an intern is giving a talk. The intern asks the editor: “What do you think of my performance?”
The editor responds: “Your performance was not satisfactory”/ “Your performance was not unsatisfactory”

Antonymic pair 10: FRIENDLY/UNFRIENDLY

Condition: Low power speaker
The professor has given some feedback to a student about his work. The professor asks the student: “What do you think of the feedback?”
The students responds: “Your feedback was not friendly”/ “Your feedback was not unfriendly”

Condition: High power speaker
A student has given some feedback to the professor about his work. The student asks the professor: “What do you think of the feedback?”

The professor responds: “Your feedback was not friendly”/ “Your feedback was not unfriendly”

Antonymic pair 11: USEFUL/USELESS

Condition: Low power speaker
At the weekly team meeting, the editor has taken notes on the current projects to send to an intern. The editor asks the intern: “How do you find my notes?”
The intern responds: “Your notes are not useful”/ “Your notes are not useless”

Condition: High power speaker
At the weekly team meeting, an intern has taken notes on the current projects to send to the editor. The intern asks the editor: “How do you find my notes?”
The editor responds: “Your notes are not useful”/ “Your notes are not useless”

Antonymic pair 12: GOOD/BAD

Condition: Low power speaker
The professor gives a talk about her latest research project. The professor asks a student: “What do you think of my talk?”
The student responds: “Your talk was not good”/ “Your talk was not bad”

Condition: High power speaker
A student gives a talk about her latest research project. The student asks the professor: “What do you think of my talk?”
The professor responds: “Your talk was not good”/ “Your talk was not bad”

Antonymic pair 13: STRONG/WEAK

Condition: Low power speaker
The professor is presenting his arguments against the new university policy on plagiarism. The professor asks a student: “What do you think of my arguments?”
The student responds: “Your arguments are not strong”/ “Your arguments are not weak”

Condition: High power speaker
A student is presenting his arguments against the new university policy on plagiarism. The student asks the professor: “What do you think of my arguments?”
The professor responds: “Your arguments are not strong”/ “Your arguments are not weak”

Antonymic pair 14: KIND/MEAN
Condition: Low power speaker
The editor is taking part in a mock questions and answers session before an intern speaks at an important conference. The editor asks the intern: “What do you think of my questions?”
The intern responds: “Your questions are not kind”/ “Your questions are not mean”

Condition: High power speaker

An intern is taking part in a mock questions and answers session before the editor speaks at an important conference. The intern asks the editor: "What do you think of my questions?"
The editor responds: "Your questions are not kind"/ "Your questions are not mean"

Antonymic pair 15: TALL/SHORT

Condition: Low power speaker

At the architecture exhibition organized by the university, the professor is presenting his model for a city tower. The professor asks a student: "What do you think of the building?"
The student responds: "The building is not tall"/ "The building is not short"

Condition: High power speaker

At the architecture exhibition organized by the university, a student is presenting his model for a city tower. The student asks the professor: "What do you think of the building?"
The professor responds: "The building is not tall"/ "The building is not short"

Antonymic pair 16: HAPPY/SAD

Condition: Low power speaker

The general assembly of the employees has approved a salary reduction for the executive directors. Later that week, the boss and an employee meet. The boss asks the employee: "What do you think about this decision?"
The employee responds: "The executive directors are not happy"/ "The executive directors are not sad"

Condition: High power speaker

The general assembly of the employees has approved a salary reduction for the executive directors. Later that week, an employee and the meet. The employee asks the boss: "What do you think about this decision?"
The boss responds: "The executive directors are not happy"/ "The executive directors are not sad"

Antonymic pair 17: LONG/SHORT

Condition: Low power speaker

The editor has put together a portfolio to apply for a grant from a journalism association. The editor asks an intern: "How do you find my publications?"
The intern responds: "Your publication list is not long"/ "Your publication list is not short"

Condition: High power speaker

An intern has put together a portfolio to apply for a grant from a journalism association. The intern asks the editor: "How do you find my publications?"
The editor responds: "Your publication list is not long"/ "Your publication list is not short"

Antonymic pair 18: POLITE/RUDE

<u>Condition: Low power speaker</u>
The editor has written an article to reply to ongoing accusations of poor fact-checking. The editor asks the intern: “How do you find my reply?”
The intern responds: “Your reply is not polite”/ “Your reply is not rude”

<u>Condition: High power speaker</u>
An intern has written an article to reply to ongoing accusations of poor fact-checking. The intern asks the editor: “How do you find my reply?”
The editor responds: “Your reply is not polite”/ “Your reply is not rude”

Antonymic pair 19: RICH/POOR

<u>Condition: Low power speaker</u>
The professor has commented on a draft paper of a student. The professor asks the student: “What do you think of my comments?”
The student responds: “The list of comments is not rich” / “The list of comments is not poor”

<u>Condition: High power speaker</u>
A student has commented on a draft paper of the professor. The student asks the professor: “What do you think of my comments?”
The professor responds: “The list of comments is not rich” / “The list of comments is not poor”

Antonymic pair 20: SATISFACTORY/FRUSTRATING

<u>Condition: Low power speaker</u>
At a staff gathering, the boss is presenting his sales pattern over the last month. The boss asks the employee: “How do you find the pattern?”
The employee responds: “The pattern is not satisfactory”/ “The pattern is not frustrating”

<u>Condition: High power speaker</u>
At a staff gathering, an employee is presenting his sales pattern over the last month. The employee asks the boss: “How do you find the pattern?”
The boss responds: “The pattern is not satisfactory”/ “The pattern is not frustrating”
