# Supplementary material for: Face Management and Negative Strengthening: The Role of Power Relations, Social Distance, and Gender
Source: Front Psychol. 2021 Sep 27;12:602977. doi: 10.3389/fpsyg.2021.602977 (PMC8502883; doi:10.3389/fpsyg.2021.602977)
Supplement: Supplementary file 2 [file Table_2.pdf]

APPENDIX B
List of stimuli for Experiment 2

For each scenario, *X* and *Y* were replaced by “Sue” and “Mary”, or vice versa, in the Female speaker condition) and by “Paul” and “John”, or vice versa, in the Male speaker condition.

Antonymic pair 1: CERTAIN/UNCERTAIN
X and *Y* work for the same company and they are very good friends [Low distance] / *X* and *Y* just started working in the same company [High distance]. *X* is negotiating a deal with an important customer. *X* asks *Y*: “What do you think of the deal?”
Y responds: “Your deal is not certain”/ “Your deal is not uncertain”

Antonymic pair 2: LUCKY/UNLUCKY
X and *Y* work for the same company and they are very good friends [Low distance] / *X* and *Y* just started working in the same company [High distance]. Following renovations, *X* has been assigned a new office room. *X* asks *Y*: “How do you find my office room?”
Y responds: “You were not lucky”/ “You were not unlucky”

Antonymic pair 3: ACCURATE/INACCURATE
X and *Y* work for the newspaper and they are very good friends [Low distance]/ *X* and *Y* just started working for the newspaper [High distance]. The newspaper prepares a special issue on the upcoming election. *X* has written a feature on the leading candidate. *X* asks *Y*: “What do you think of the feature?”
Y responds: “Your feature is not accurate”/ “You feature is not inaccurate”

Antonymic pair 4: HAPPY/UNHAPPY
X and *Y* work for the same company and they are very good friends [Low distance] / *X* and *Y* just started working in the same company [High distance]. At a meeting with an important client, *X* has presented her/his new marketing strategy. *X* asks *Y*: “How did you find the client's reaction?”
Y responds: “The client was not happy”/ “The client was not unhappy”

Antonymic pair 5: INTERESTING/UNINTERESTING
X and *Y* study at the same university and are very good friends [Low distance]/ *X* and *Y* just started studying at a new university [High distance]. In history class, *X* has shown a video about the French Revolution. *X* asks *Y*: “What do you think of the video?”
Y responds: “The video is not interesting”/ “The video is not uninteresting”.

Antonymic pair 6: FAIR/UNFAIR
X and *Y* work for the same company and they are very good friends [Low distance] / *X* and *Y* just started working in the same company [High distance]. At a staff gathering in the factory meeting room, *X* has presented the work schedule she/he prepared for that day. *X* asks *Y*: “How do you find the schedule?”
Y responds: “Your schedule is not fair”/ “Your schedule is not unfair”

Antonymic pair 7: POLITE/IMPOLITE

X and *Y* work for the newspaper and they are very good friends [Low distance]/ *X* and *Y* just started working for the newspaper [High distance]. The newspaper would like to interview the leading candidate of the upcoming election. *X* writes an email to request a meeting. *X* asks *Y*: "What do you think of my email?"
Y responds: "Your email is not polite"/ "Your email is not impolite"

Antonymic pair 8: POSSIBLE/IMPOSSIBLE

X and *Y* work for the same company and they are very good friends [Low distance] / *X* and *Y* just started working in the same company [High distance]. *X* is behind with the working schedule. The prototype needs to be delivered to the client by the end of the week. *X* asks *Y*: "What do you think about extending the deadline?"
Y responds: "An extension is not possible"/ "An extension is not impossible"

Antonymic pair 9: SATISFACTORY/UNSATISFACTORY

X and *Y* work for the newspaper and they are very good friends [Low distance]/ *X* and *Y* just started working for the newspaper [High distance]. At the yearly meeting of the press association, *X* is giving a talk. *X* asks *Y*: "What do you think of my performance?"
Y responds: "Your performance was not satisfactory"/ "Your performance was not unsatisfactory"

Antonymic pair 10: FRIENDLY/UNFRIENDLY

X and *Y* study at the same university and are very good friends [Low distance]/ *X* and *Y* just started studying at a new university [High distance]. *X* has given some feedback to *Y* about her/his work. *X* asks *Y*: "What do you think of the feedback?"
Y responds: "Your feedback was not friendly"/ "Your feedback was not unfriendly"

Antonymic pair 11: USEFUL/USELESS

X and *Y* work for the newspaper and they are very good friends [Low distance]/ *X* and *Y* just started working for the newspaper [High distance]. At the weekly team meeting, *X* has taken notes on the current projects to send to *Y*. *X* asks *Y*: "How do you find my notes?"
Y responds: "Your notes are not useful"/ "Your notes are not useless"

Antonymic pair 12: GOOD/BAD

X and *Y* study at the same university and are very good friends [Low distance]/ *X* and *Y* just started studying at a new university [High distance]. *X* gives a talk about her/his latest research project. *X* asks *Y*: "What do you think of my talk?"
Y responds: "Your talk was not good"/ "Your talk was not bad"

Antonymic pair 13: STRONG/WEAK

X and *Y* study at the same university and are very good friends [Low distance]/ *X* and *Y* just started studying at a new university [High distance]. *X* is presenting her/his arguments against the new university policy on plagiarism. *X* asks *Y*: "What do you think of my arguments?"

Y responds: “Your arguments are not strong”/ “Your arguments are not weak”

Antonymic pair 14: KIND/MEAN
X and *Y* work for the newspaper and they are very good friends [Low distance]/ *X* and *Y* just started working for the newspaper [High distance]. *X* is taking part in a mock questions and answers session before *Y* speaks at an important conference. *X* asks *Y*: “What do you think of my questions?”
Y responds: “Your questions are not kind”/ “Your questions are not mean”

Antonymic pair 15: TALL/SHORT
X and *Y* study at the same university and are very good friends [Low distance]/ *X* and *Y* just started studying at a new university [High distance]. At the architecture exhibition organized by the university, *X* is presenting her/his model for a city tower. *X* asks *Y*: “What do you think of the building?”
Y responds: “The building is not tall”/ “The building is not short”

Antonymic pair 16: HAPPY/SAD
X and *Y* work for the same company and they are very good friends [Low distance] / *X* and *Y* just started working in the same company [High distance].The general assembly of the employees has approved a salary reduction for the executive directors. Later that week, *X* and *Y* meet. *X* asks *Y*: “What do you think about this decision?”
Y responds: “The executive directors are not happy”/ “The executive directors are not sad”

Antonymic pair 17: LONG/SHORT
X and *Y* work for the newspaper and they are very good friends [Low distance]/ *X* and *Y* just started working for the newspaper [High distance]. *X* has put together a portfolio to apply for a grant from a journalism association. *X* asks *Y*: “How do you find my publications?”
Y responds: “Your publication list is not long”/ “Your publication list is not short”

Antonymic pair 18: POLITE/RUDE
X and *Y* work for the newspaper and they are very good friends [Low distance]/ *X* and *Y* just started working for the newspaper [High distance]. *X* has written an article to reply to ongoing accusations of poor fact-checking. *X* asks *Y*: “How do you find my reply?”
Y responds: “Your reply is not polite”/ “Your reply is not rude”

Antonymic pair 19: RICH/POOR
X and *Y* study at the same university and are very good friends [Low distance]/ *X* and *Y* just started studying at a new university [High distance]. *X* has commented on a draft paper of *Y*. *X* asks *Y*: “What do you think of my comments?”
Y responds: “The list of comments is not rich” / “The list of comments is not poor”

Antonymic pair 20: SATISFACTORY/FRUSTRATING

X and *Y* work for the same company and they are very good friends [Low distance] / *X* and *Y* just started working in the same company [High distance]. At a staff gathering, *X* is presenting her/his sales pattern over the last month. *X* asks *Y*: "How do you find the pattern?" *Y* responds: "The pattern is not satisfactory"/ "The pattern is not frustrating"
